# Hybrid Organic/Inorganic Nanocomposites for Photovoltaic Cells

**DOI:** 10.3390/ma7042747

**Published:** 2014-04-02

**Authors:** Ruchuan Liu

**Affiliations:** College of Physics, Chongqing University, No. 55 Daxuecheng South Road, Shapingba District, Chongqing 401331, China; E-Mail: phyliurc@cqu.edu.cn; Tel.: +86-136-383-70036; Fax: +86-23-6567-8362

**Keywords:** solar cell, semiconductor, conjugated polymer, nanostructure, photovoltaic, charge separation, interface, nanocomposites

## Abstract

Inorganic/organic hybrid solar cells have attracted a lot of interest due to their potential in combining the advantages of both components. To understand the key issues in association with photoinduced charge separation/transportation processes and to improve overall power conversion efficiency, various combinations with nanostructures of hybrid systems have been investigated. Here, we briefly review the structures of hybrid nanocomposites studied so far, and attempt to associate the power conversion efficiency with these nanostructures. Subsequently, we are then able to summarize the factors for optimizing the performance of inorganic/organic hybrid solar cells.

## 1. Introduction

In searching for renewable energy sources, solar cells represent a class of promising candidates. In the past decades, a tremendous amount of research work has been carried out in the relevant fields and many types of photovoltaic cells have been developed and tested. So far, all of these photovoltaic cells are still a long way from satisfying the various requirements demanded, and many critical issues remain or are being studied. Most of the issues being studied target the key goal, the improvement of the power conversion efficiency of the photovoltaic cells.

Very many types of photovoltaic materials, e.g., inorganic semiconductor nanoparticles, silicon, organic dyes, conducting polymers, C_60_ derivatives, and graphene *etc*. as well as their combinations have already been attempted. Each type of these materials has its merits and drawbacks. Organic conducting polymers with a variety of chemical structures, for instance, have promising properties, such as easy processing, possible recyclability, relatively low cost, scalability and applicability as sustainable materials. Inorganic semiconductors, on the other hand, possess better electronic properties, e.g. a high dielectric constant, a high charge mobility and thermal stability *etc*., while their nanoparticles exhibit enhanced electronic, photo-conducting and luminescent properties. To take advantage of both materials, hybrid nanocomposites of inorganic semiconductors and conducting polymers are of great interest, especially as candidates for photovoltaic-cell materials, where the combined absorption band of both materials can also better harvest sun light. With this concept, conducting polymers have been combined with different inorganic semiconductors. In addition, photo-induced charge separation mainly takes place at the interfaces between inorganic semiconductors and conducting polymers in these hybrid materials, where electrons are injected from the conducting polymers into inorganic semiconductors and holes remain in the polymers. Actually, this interfacial charge separation can to some extent prevent the recombination of separated electrons and holes. The reviews by Saunders *et al*. [1], Zhou *et al*. [2] and Wright *et al*. [3] have given a fairly complete picture of this concept of hybrid solar cells and have summarized a full list of issues, especially many experimental details, targeting a better power conversion efficiency in these hybrid systems. So far, a lot of studies to find good combinations of inorganic/organic materials and optimal hybrid architectures have been attempted, and significant progress has been achieved in recent years. However, the power conversion efficiency has not yet caught up to the level that the dye-sensitized photovoltaic materials achieved, ~12%. Nevertheless, we believe that there is still room for improvement of the hybrid organic/inorganic photovoltaic materials. For example, good combinations of the paired components and well-designed hybrid structures should be among the ways of achieving this target.

In this brief review, we look into recent progress witnessed in the field of hybrid organic/inorganic photovoltaic materials for solar cells from the physical point of view and focus on their architectural designs. By comparing the various pairs of organic/inorganic components and nano-structures proposed, we can summarize factors affecting their photovoltaic conversion efficiency and architectures achieved for these hybrid systems in order to clarify the relationship between structures and underline the physical mechanism therein, and then outline guidelines for future improvement.

## 2. Charge Separation at the Interfaces between Inorganic Semiconductors and Conducting Polymers

**Figure 1.**
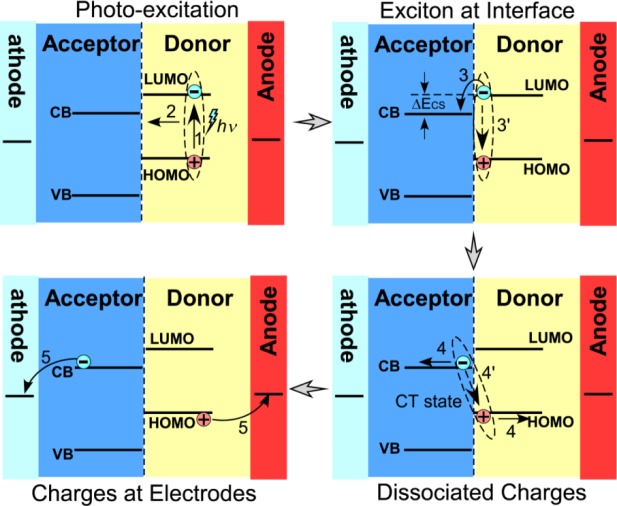
A scheme of the charge separation process at the Donor:Acceptor interface in a hybrid solar cell. The major photovoltaic steps include: photo-excitation into excitons (**1**), excitons migration to interfaces (**2**), charge transfer from the donor to the acceptor at the interface (**3**), charge migration to electrodes (**4**) and charge injections into electrodes. There are also competition processes, which can reduce the photovoltaic conversion efficiency, including the recombination of the excitons in the donor and separated charges at the interfaces.

The interfacial charge separation is one key step in the photovoltaic processes of the hybrid materials. However, factors controlling the interfacial charge separation are not fully understood. It is commonly believed that a photogenerated exciton dissociates to generate a bound complex with charge transfer character at a donor:acceptor heterojunction and then separates into fully dissociated charge carriers [4–6] as illustrated in Figure 1. Here, the efficiency of charge generation depends on whether the interfacial charge separation can successfully compete with the recombination of the bound charge transfer complex and with the energy transfer to singlet or triplet excitons of either component. During this process, factors, such as the interface area, the nominal interfacial driving energy (the differences in energy level across the interface), the morphology of the interfaces/domains, the interfacial layer/molecules/bridges, the crystallinity of the components, the mixture homogeneity, and the migration of separated charges from the interfaces, can influence the charge separation rate.

Various combinations of inorganic semiconductors and organic conducting polymers can be candidates for hybrid photovoltaic materials. From the electron acceptors, CdS [7–19], CdSe [20–46], CdTe [38,47–50], PbS [51–55], PbSe [56–60], ZnO [33,61–85] and TiO_2_ [13,86–102] *etc*., have been attempted so far, while more choices are available for conducting polymers. Within these hybrid systems, the charge generation is fundamentally governed by the energy-level alignment at the donor/acceptor interfaces, while the photocurrent in the final device depends on the appropriate energy level alignment at the electrode interfaces. Therefore, it is critical to have the optimal configuration by properly choosing the hybrid combinations together with the electrodes. So far, experimental measurements [67,102–104] and theoretical calculations [62,105,106] of the energy level alignment have been carried out for a few such combinations, e.g., hybrids of P3HT/ZnO and P3HT/TiO_2_, especially in comparison of the LUMO of the polymer and the conducting band of the inorganic semiconductor. Interestingly, Scharber *et al*. performed a comprehensive analysis of the energy level alignment in a series of composites of [6,6]-phenyl-C61-butyric acid methyl ester (PCBM) and various conducting polymers, and found an empirical linear correlation between the HOMO of the polymer and the open-circuit voltage [107]. Their contour plot for power conversion efficiency predicts that 10% is theoretically achievable for the series of hybrid photovoltaic systems. It is reasonable to believe that other hybrid photovoltaic systems follow a similar relationship between energy level alignment and optimal power conversion efficiency, but the achievable efficiency can vary. More experiments are still needed to find out good combinations of such hybrid systems.

Bansal *et al*. demonstrated that the dissociation of photogenerated polaron pairs at the heterojunctions of CdS and several conducting polymer nanocomposites is assisted by the presence of crystalline electron acceptor domains [6]. In this case, the crystallinity and larger size (>1 nm) of CdS domains can encourage delocalization of the geminate pair state and attenuate the required interfacial driving energy. Oosterhout *et al*. suggested that the morphology of the hybrid system is important [108], and the inorganic component therein improves the thermal stability. In their experiments, the internal quantum efficiency of the hybrid P3HT/ZnO nanocomposites increased with the thickness of the hybrid layer until the optimal layer thickness, which was significantly larger than 100 nm. However, the morphology analysis indicated that larger domains of P3HT and ZnO, and larger distance (>5 nm) between them in the thinner sample likely led to the lower internal quantum efficiency. Our recent study, using scanning electrostatic potential microscopy to directly monitor the interfacial charge separation in the hybrid nanocomposites of PPV and TiO_2_, showed that the holes were only able to travel around 20 nm away from the interfaces after charge separation [109]. These results suggested that there should be an optimal domain size for the hybrid nanocomposites to maximize the efficiency of photo-induced charge separations; in turn the structural design of the hybrid photovoltaic system is critical.

## 3. The Nano Architecture of Hybrid Photovoltaic Materials

**Figure 2.**
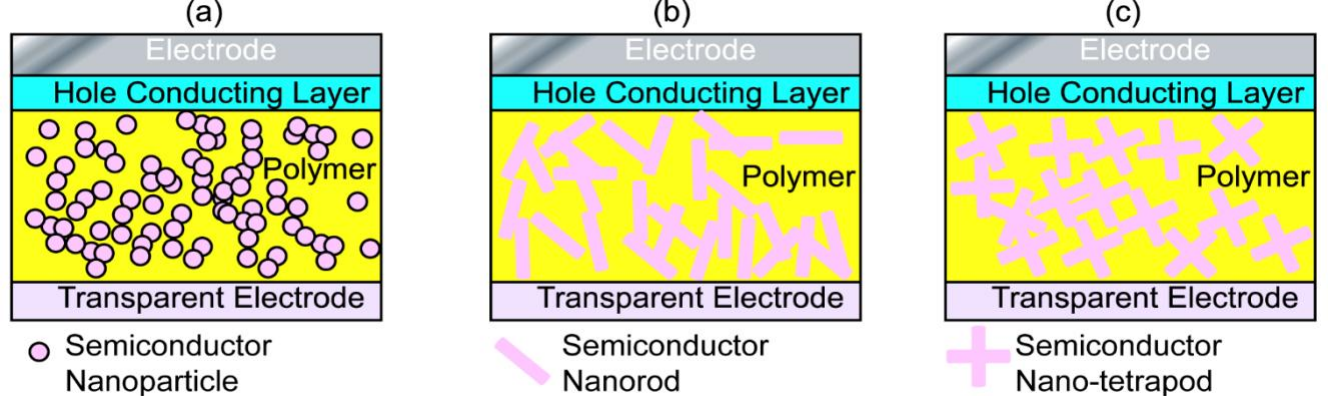

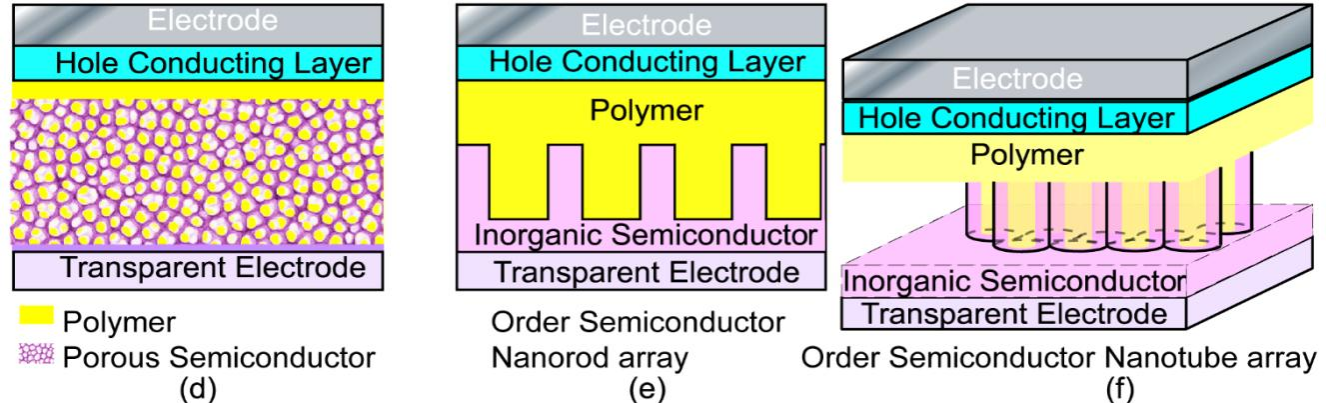
Various nano architectures of solar cell materials. (**a**) Blend of semiconductor nanoparticles and conducting polymer films; (**b**) blend of semiconductor nanorods and conducting polymer films; (**c**) blend of semiconductor nano-tetrapods and conducting polymer films; (**d**) conducting polymer immersed in porous semiconductor nano-network; (**e**) blend of semiconductor nanorods arrays and conducting polymer films; and (**f**) blend of semiconductor nanotube arrays and conducting polymer films.

Because the interfacial charge separation is the critical step in the whole photovoltaic process, the larger the interfaces, the more the opportunity for the excitons to reach the interfaces, and probably the higher the conversion efficiency. Therefore, most of the hybrid photovoltaic materials that have been studied are nano-structured composites. There are many types of nanostructures, including particles, rods, tubes, tetrapods, sheets, needles, and porous network, *etc*., and the way to mix the components together can be either disordered or ordered as shown in Figure 2.

### 3.1. Quantum Dots/Nanocrystal Based Systems

Quantum dots/nanocrystals of inorganic semiconductors based systems for solar cells have been extensively studied over the past decades [22,44,110–115], because quantum dots (QDs)/nanocrystals are expected for a larger surface area than bulk materials. In blending with a conducting polymer, these hybrid systems can also take advantage of the large-area solution processing. In addition, the quantum size effect leads to a tunable energy gap [54] of QDs to allow energy level matching between the components in the solar cell systems, while a better coverage of the spectrum of sunlight is also expected.

The blending of QDs/nanocrystals with conducting polymers is not as simple as just mixing and coating. The procedure details of blending influence the final nano-structure and hybrid interfaces, which can determine the photovoltaic processes therein. There is still considerable room for optimization of their power conversion efficiency.

Because capping is important in the synthesis of QDs/nanocrystals and different cappings are known to alter the contact between QDs and conducting polymers, several capping strategies have emerged in the literature: the ligand exchange [110,116,117], the use of novel surfactants [118,119], the synthesis of QDs with thermally cleavable ligands [120], the synthesis of QDs in solutions of conducting polymers [52,121], the polymerization of conducting polymers on the surface of QDs [122], and the deposition of a conducting polymer containing a soluble precursor of the QDs [123]. More details on the role of capping ligands/conducting polymers can be found in recent reviews [124–128]. Reynolds *et al*. reported *in situ* growth of CdS QDs from the precursor in a P3HT polymer film, and found that the solar cells from the blends of P3HT and *in situ* growth of CdS QDs displayed much higher open-circuit voltage (*V*_OC_) and short-circuit current (*J*_SC_) than that from the blend of P3HT and CdS QDs capped by oleic acid or hexylamine. They proposed that the difference is due to the morphology/interfaces, where the *in situ* grown CdS QDs formed an interconnected layer, in sharp contrast to the discrete, unaggregated nanocrystals in the pre-synthesized and capped QDs [123]. This indicates a physical fact that the contact between the conducting polymer and the QDs, as well as between QDs themselves, is essential for the photovoltaic performance, because interfacial charge separation and charge migration throughout the photovoltaic process can eventually be affected by these contacts. Similarly, the solution conditions during QD synthesis and QDs/polymer mixing, and later annealing of the blend films can also influence the phase separation, the interfacial conditions and in turn the power conversion efficiency.

### 3.2. Nanorods/Nanotetrapods Based Systems

Nanorods/nanotetrapods of inorganic semiconductor based systems have also received considerable interest.[21,77,129–131] Though nanorods/nanotetrapods are not so easy to synthesize as nanoparticles/QDs and the processes of the corresponding hybrid systems require more harsh conditions, the larger surface area to volume ratio of nanorods (Figure 2b)/nanotetrapods (Figure 2c), the smaller number of interparticle hops necessary for electrons to leave the device, and thus the better connected paths are suggested as enhancing the power conversion efficiency [132–134]. Sun *et al*. [132] compared blends of branched CdSe nanoparticles and conjugated polymers with similar blends of nanorods/polymer, and found a significant increase in external quantum efficiency when branched nanoparticles were used. Lee *et al*. [133] further compared hybrid solar cells based on long-armed nanotetrapods and short-armed nanotetrapods, and found that the nanotetrapods with longer arms lead to greater short-circuit current density and fill factor than those with short arms. They suggested that the higher charge transport efficiency in long-armed nanotetrapods increases both the quantum efficiency and the fill factor, and thus leads to better power conversion efficiency in the hybrid systems. Morphologically, the hybrid system based on long-armed nanotetrapods presents a longer percolation pathway and larger domains of P3HT, where more favorable hole drift and diffusion can be expected. This can minimize exciton recombination events along the charge transport pathways. Of this type of hybrid system, Dayal *et al*. [41] reported a high power conversion efficiency of 3.13% in the nanotetrapods of a CdSe based system, where a low band gap (~1.4 eV) conjugated polymer PCPDTBT was incorporated. Soci *et al*. [135] claimed that a band gap of 1.5 eV was optimum for conjugated polymers in hybrid solar cells.

Another interesting nanostructure is based on inorganic semiconductor quantum dots and nanorods of the conducting polymer. Ren *et al*. [136] used solvent-assisted chemical grafting and ligand-exchange to synthesize P3HT nanowires and decorated them with CdS QDs, and the power conversion efficiency was as high as 4.1%. The high efficiency in this hybrid system can be attributed to the enhanced charge collection efficiency due to continuous percolated nanomorphology in both components, where more efficient transport pathways for both electrons and holes are available. In addition, the charge separation efficiency may also be enhanced between P3HT nanowires and CdS QDs. This is an interesting hybrid structure, as an invert to the nanorods of the inorganic semiconductor based system discussed above. In both nanorod based systems, the long nanorods provide better pathways for charge transport and reduce the need of inter-particle hops for charge carriers.

### 3.3. Nanoporous Inorganic Semiconductor-Based Hybrid Systems

There are other various choices of hybrid structures for the disordered nanocomposite system. Among them, the nanoporous structure (Figure 2d) is of great interest, and many attempts have been made based on porous inorganic semiconductors, such as TiO_2_ [137–139], CdS [140], ZnO [141], CdSe(ZnS) [142] *etc*. De Freitas *et al*. compared films of P3HT:aerogel of CdSe(ZnS) with films of P3HT:quantum dots with a similar ratio of each component, where the films of the P3HT:aerogel showed increased photocurrent and charge generation. This was attributed to the existence of a more interconnected network, which extended the lifetime of the photoinduced charge separated state and reduced the chance of recombination of the separated charges [142]. The idea of an interconnected network of the hybrid components in the systems is the same as using branched inorganic nanoparticles when mixing with conducting polymers. However, the performance of this type of photovoltaic cell achieved is still not as good as expected, because there are still other factors that can limit the power conversion efficiency, e.g., poor contact between the porous semiconductor and the conducting polymer. Therefore, improvements are still eagerly anticipated for this type of photovoltaic material, particularly in obtaining better interfaces between the polymer and the porous inorganic base.

### 3.4. Ordered Nanorod Array (NRA) Based System

In the above hybrid systems, the very large interfacial area achieved between the inorganic semiconductor and the conjugated polymer can lead to efficient dissociation of excitons into holes and electrons. However, the charge carrier recombination inevitably takes place here or there during the charge transport through the random networks of the material, and eventually limits the PCE. From the above discussion, it is quite clear that improved pathways for charge transport can greatly help the performance of the solar cells. In this regard, ordered hybrid solar cells are proposed as a promising structure, in which the donor and acceptor materials are interdigitated at the nanometer scale, providing more direct pathways for charge transport and avoiding the need for inter-nanoparticle hops. The ordered nanorod array based systems have been proposed and examined in many research laboratories [9,61,143–156].

Lee *et al*. [157] compared a hybrid system based on a vertically aligned 180 nm ZnO NRA with a bilayer one, and found an ~2.7× enhancement of the short-circuit current. Similarly, Choi *et al*. [144] reported a better PCE in a photovoltaic device based on a ZnO nanostructure length of 100 nm than in an equivalent device without ZnO nanorods. However, the achieved power conversion efficiency of bicomponent hybrid systems (ZnO NRA/P3TH) is not as good as those based on disordered nanorods. The introduction of third party molecule/materials at the interfaces can greatly enhance the performance of the final heterojunctions, e.g., using ZnO/CdS-core/shell heterostructures [158], adding a third component of a small dye molecule [141,147,159] or [6,6]-phenyl-C_61_-butyric acid methyl ester (PCBM) [156]. Compared with the original bicomponent hybrid system of the conjugated polymer/inorganic semiconductor NRAs, an increment in the power conversion efficiency of the multicomponent NRA based system was found. In the case of ZnO NRA/P3HT system adding PCBM, the increment of the PCE is more significant, but it is still far below the best performance found for the PCBM polymer solar cell, which is now over 10% [160]. Therefore, it is hard to draw a conclusion that the increment of PCE is due to adding a third component to these solar cells. Instead, it may be just a “half pure” PCBM/conducting polymer hybrid system. Nevertheless, the maximal PCE for these ordered NRA hybrid systems is only comparable to that of their equivalent disordered systems. Though the interfacial area in the ordered NRA hybrid system is larger than that in the plane bilayer hybrid system, it is much smaller than the disordered nanorods based equivalent. The impact of the relatively limited interfacial area on the PCE counteracts that of the better pathways for charge transport.

### 3.5. Ordered Nanotube Array (NTA) Based System

**Figure 3.**
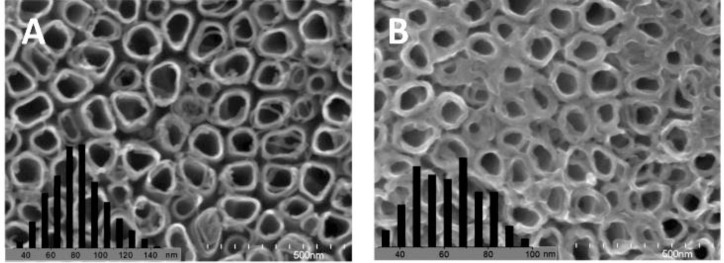

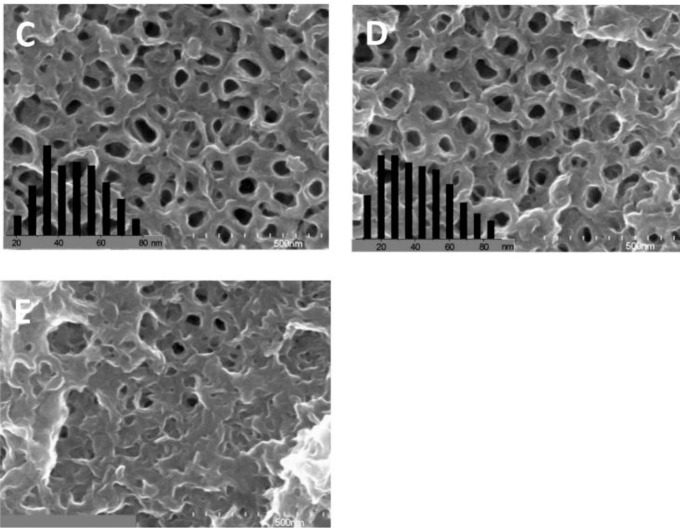
SEM images of electrochemical polymerization of PEDOT in TiO_2_ nanotube arrays: (**A**) initial TiO_2_ nanotube arrays; (**B**–**E**) different stages of the polymerization of EDOT on the substrate. Reproduced with permission from [169], Copyright 2010, American Chemical Society.

To increase the interfacial area in the ordered nano array hybrid system while keeping the effective charge transport pathways, ordered nanotube arrays as shown in Figure 2f were used. [15,88,90,161–172] However, experimentally those nanotube arrays were not as perfect as displayed in Figure 2f, e.g., a typical FESEM of TiO_2_ nanotube arrays as shown in Figure 3 [170], so improvements in the chemical/electrochemical synthesis are needed. Nevertheless, a better performance of the ordered nanotube array based system is expected because of the larger interfaces than their nanorod equivalents. However, similar to the ordered nanorod array based system, the PCE for TiO_2_ NTA based systems reported by different groups varied by more than one order of magnitude (Table 1). [15,88,90,161–169] Again, the introduction of third party molecules/materials at the interfaces can greatly increase the PCE of these hybrid systems. Li *et al*. [15] reported that the PCE of the hybrid system Au/P3HT/CdS-NTA/FTO has a ~4 fold increase in the PCE compared with the hybrid system Au/P3HT/NTA/FTO. Besides the CdS shell, quite a few dyes [164,165,169] and PCBM[88,161,163] have also be incorporated into the P3HT/TiO_2_-NTA system, and in most cases the PCE can exceed 1%. For example, Shankar *et al*., reported a PCE of 4.1% in a hybrid system of P3HT-PCBM-TiO_2_ [163]. Interestingly, to improve the infiltration of conducting polymers into NTAs and their contact, *in situ* electrochemical polymerization of polythiophene was utilized, but the PCE of 1.46% obtained was not as good as expected. Thus, although this improvement may not be significant, it should still be the right direction to get better interfaces between inorganic and organic components in these ordered hybrid systems. However, there are still quite a few other factors that cannot be neglected.

## 4. Important Factors in the Performance of Photovoltaic Materials

Some representative hybrid systems as described above with reported power conversion efficiency are summarized in Table 1. The best performance achieved among these hybrid systems is one combining P3HT and nanotube arrays of TiO_2_ [163]. However, several hybrid systems of other types of structures can also be very close in performance [136,156]. From the power conversion efficiency among these systems as listed in Table 1, we can confirm the influence of the factors discussed in the previous section on the charge separation processes. However, their importance varies, and we suggest that the following factors are critical and need optimization for the improvement of these hybrid systems. First, the charge transportation along both components can determine the current density and so affect the final performance. A continuous network of the components, e.g., long nanorods and branched nanorods, can greatly reduce the need for charge carriers to hop between nanoparticles during their transportation to the electrode, so that the nanorods and nanotube array-based hybrid systems showed overall better performances in reports. However, the carrier transportation in conducting polymers is especially one of the bottlenecks in these hybrid systems, and the improvement on the quality of the conducting polymer network can greatly enhance the power conversion efficiency of the final system as reported by Ren *et al*. [136] Second, the interfacial area and contact between inorganic and organic components determine the charge separation efficiency. The nanotube array-based systems show on average better performance than nanorod array-based systems because the surface area of nanotubes is larger than that of nanorods of similar sizes. At the same time, a 3D network of porous structure-based systems, although it would be expected to enhance the carrier transportations, is not so successful, which is likely due to the fact that the inorganic-organic interfaces are far away from good contacts. Third, the energy-level alignment at the interfaces is also very critical for optimal hybrid systems, so their components should be carefully chosen. According to our understanding from the literature, the following guidelines can be stated for the improvement of hybrid photovoltaic systems of inorganic semiconductors/conducting polymers.

First, the right combination of inorganic and organic semiconductors should be chosen. For this purpose, the LUMO of the conducting polymer needs to be aligned with the conducting band of the inorganic semiconductor, or the band-gap of the inorganic semiconductor can be tuned by quantum confinement effect. In addition, an optimal band-gap of ~1.5 eV is suggested for conducting polymers in the hybrid system. This is physically sound and reasonable, because such a band-gap may balance a large enough absorption of sunlight (thus a good photocurrent) and a high enough output photo-voltage.

Second, nanostructures should be used to provide a large interface for the enhancement of the charge separation process, but at the same time the structural design has also to consider the connection in each component to facilitate charge transportation. Thus, long nanorods/nanotubs, branched nanopods, or a porous network may be utilized, and to accelerate the charge transportation, an ordered nanorod/nanotube array can be considered.

Third, good contact between organic and inorganic components should also be considered as a prerequisite due to the improvement in charge separation at the contact interface. Here, optimal conditions for blending of components (e.g., *in situ* synthesis of mixed components and annealing *etc*.), exchanging of the capping group of the inorganic nanoparticles, and incorporation of small organic dye molecules can be used. A detailed review in this respect on the experimental conditions of synthesis was given by Zhou *et al*. [177]. In addition, we think that the hybrid system is not necessary to be bi-component and suggest that another inorganic or organic semiconductor can be introduced as a third component to enhance the light absorption, to optimize the energy-level alignment and to improve the contacts between components. For instance, PCBM and its derivatives have been used as the third component, and improvements in PCE have been reported, which are however still not comparable to that of the PCBM/derivative and conducting polymer hybrid systems. The reason may be that the introduction of a third component may also break the continuity of charge transportation along each component. Good comparisons of these two types of hybrid systems have been reviewed in detail by Saunders [178] and Reiss *et al*. [124] However, inorganic semiconductors as one of the components in these hybrid systems have their unique advantages too, and a better PCE can be expected. Nevertheless, in future architectural designs of these hybrid solar cells, the negative effect, *i.e.*, the breakage of the continuity of the charge transportation pathway, on the introduction of multi-components should be minimized.

Fourth, a nano-structured network of a conducting polymer in the hybrid system may be more helpful, because the mobility of holes in conducting polymers is much more limited than that of electrons in inorganic semiconductors.

Last but not least, the inorganic/organic blending layer in the photovoltaic system is more or less symmetrical, so physically, the random diffusion of the charge carriers will give them similar chances to move to both electrodes and thus decrease the effective power conversion of sunlight. Therefore, if the structural design can break or limit the symmetry in this blending layer, the power conversion efficiency of the resulted hybrid system can also be improved. In the work of Liu *et al*. [174], the highest PCE (~5.5%) of this type of solar cell has been reported so far, the vertical phase segregation of PDTPBT and PbS*_x_*Se_1−_*_x_* has been claimed as one of the reasons for the PCE reached. In fact, this vertical phase segregation resulted in a nanostructure more or less breaking the symmetry of the components usually seen in other reports, although they did not claim the effect from symmetry breakage.

## 5. Conclusions

Here, we briefly reviewed recent progress on hybrid photovoltaic systems of inorganic semiconductors and organic conducting polymers. We focused on nanostructures and their impact on the photovoltaic performance of these hybrid materials. From the results from the literature, we summarized and clarified the critical factors for optimization of the hybrid inorganic/organic semiconductors. Finally, based on our knowledge, we suggested a few guidelines for structural designs of these hybrid photovoltaic systems in order to enhance their power conversion efficiency.

## Figures and Tables

**Table 1.** Photovoltaic materials, structures and efficiency.

Components	Basic Structure	Capping	Power Conversion Efficiency	Ref.	Extra
ITO/PEDOT:PSS/MDMO-PPV:PbS QDs/Al	Quantum dots in polymer	MDMO-PPV	0.013%	[52]	–
ITO/PEDOT:PSS/MDMO-PPV:SnS_2_/ZnO/Al	SnS_2_ nanoparticles in polymer	pyrindine OLA	0.31% 0.204%	[116]	Annealing –
MEH-PPV:CdSe	Nanocrystals Nanocrystals	Oleic acid	0.85% 2.8%	[44]	–
PCPDTBT:CdSe	Nanocrystals/nanorods	–	3.6%	[111]	–
PSiF-DBT:CuInS_2_	Nanocrystals	–	2.8%	[173]	–
PDTPBT:PbS*_x_*Se_1−_*_x_*	Nanocrystals	–	5.5%	[174]	Vertical segregation
PPV:CdTe	Nanocrystals	–	4.76%	[175]	–
P3HT:GaN	QDs	–	0.14%	[113]	–
P3HT:PCBM:CuInS_2_	QDs	–	2.76%	[115]	–
P3HT:ZnO	Nanorods	–	0.22%	[129]	–
P3HT:ZnO/squaraine			1.02%		–
P3HT:ZnO (Li doped)	Nanorods	–	0.37%	[77]	–
PCPDTBT:CdSe	Nanorods	–	5.2%; 4.7%	[176]	–
OC_1_C_10_-PPV:CdSe	Branched nanoparticles	–	1.8%	[132]	–
ITO/PEDOT:PSS/P3HT:CdSe/LiF:Al	Nanotetrapods	–	0.8% 1.12%	[133]	5 nm 10 nm
P3HT:CdSe	Nanorods	–	1.7%	[134]	–
PCPDTBT/CdSe	Nanotetrapods	–	3.13%	[41]	–
P3HT:ZnO	3D nanonetwork	SQ2	0.69%	[141]	–
P3HT/CdS	Nanoporous	–	0.06%	[140]	–
P3HT:N719/CdS			1.31%		–
P3HT:CdS	QDs+nanowire of P3HT	–	4.1%	[136]	–
P3HT:ZnO	Ordered nanorod array of ZnO	–	0.76%	[61]	–
MEH-PPV:ZnO	Ordered nanorod array of ZnO	–	0.61%	[143]	–
P3HT/PCBM:ZnO	Ordered nanorod array of ZnO	–	2.4%	[144]	–
P3HT/Eosin-Y:ZnO P3HT:ZnO	Nanorod array of ZnO	–	0.39% 0.05%	[147]	–
PEDOT:PSS/ZnO/Rods/P3HT:PCBM/MoO_3_/Al	Nanorod array of ZnO	–	1.6%	[149]	–
PEDOT:PSS/ZnO/P3HT:PCBM/MoO_3_/Al	–		1.3%		
ITO/ZnO/Rods/P3HT:PCBM/MoO_3_/Al	Nanorod array of ZnO		2.3%		
ITO/ZnO/P3HT:PCBM/Ag	Nanorod array of ZnO	–	2.7%	[150]	–
ITO/ZnO/P3HT/VO*_x_*/Ag	Nanorod array of ZnO	–	3.7%	[151]	Depend on length
P3HT:PCBM/ZnO	Nanorod array of ZnO	– N719	1.16% 2.0%	[152]	–
PET/ITO/ZnO thin film/ZnO nanorods/P3HT:PCBM/Ag	Nanorod array of ZnO	–	1.78%	[153]	–
ITO/ZNAs/P3HT:PCBM/MoO_3_/Ag	Nanorod array of ZnO	–	1.11%	[155]	–
ITO/ZnO/ZnO nanorod/P3HT:PCBM/MoO_3_/Ag	Nanorod array of ZnO	–	4.07%	[156]	–
ITO/TiO_2_/P3HT:PCBM/V_2_O_5_/Al	Nanotube array of TiO_2_	–	2.71%	[161]	–
Polythiophene/TiO_2_	Nanotube array of TiO_2_	–	1.46%	[90]	–
FTO/PEDOT:PSS/P3HT:PCBM/TiO_2_	Nanotube array of TiO_2_	–	4.18%	[163]	–
FTO/PEDOT:PSS/P3HT:PCBM/TiO_2_	Nanotube array of TiO_2_	–	4.1%	[88]	–
FTO/PEDOT:PSS/P3HT:SQ-1/TiO_2_	Nanotube array of TiO_2_	–	3.8%	[165]	–
FTO/TiO_2_/CdS-TNA/P3HT/Au	Nanotube array of TiO_2_ & CdS QDs	–	3.26%	[15]	–
FTO/TiO_2_/CdS-TNA/P3HT/Au	Nanotube array of TiO_2_ & CdS shell	–	1.16%	[167]	–

## Abbreviations

P3HT: poly(3-hexylthiophene-2,5-diyl)
LUMO: lowest unoccupied molecular orbital
PCBM: [6,6]-phenyl-C_61_-butyric acid methyl ester
HOMO: highest occupied molecular orbital
PPV: poly(p-phenylene vinylene)
QD: quantum dot
PCPDTBT: poly[2,6-(4,4-bis-(2-ethylhexyl)-4*H*-cyclopenta [2,1-*b*,3,4-*b*′]dithiophene)-*alt*-4,7(2,1,3-benzothiadiazole)]
NRA: nanorod array
NTA: nanotube array
PCE: power conversion efficiency
FTO: fluorine-doped tin oxide
PSS: poly(3,4-ethylenedioxythiophene) poly(styrenesulfonate)
MDMO-PPV: poly[2-methoxy-5-(3′,7′-dimethyloctyloxy)-1,4-phenylenevinylene]
MEH-PPV: poly[2-methoxy-5-(2-ethylhexyloxy)-1,4-phenylenevinylene]
OC_1_C_10_-PPV: poly(2-methoxy-5-(3′,7′-dimethyl-octyloxy)-p-phenylenevinylene)
N719: di-tetrabutylammonium *cis*-bis(isothiocyanato)bis(2,2′-bipyridyl-4,4′-dicarboxy lato)ruthenium(II)
ITO: indium tin oxide
PET: polyethylene terephthalate

## References

[1] Saunders B.R., Turner M.L. (2008). Nanoparticle-Polymer Photovoltaic Cells. Adv. Colloid Interface Sci.

[2] Zhou Y.F., Eck M., Kruger M. (2010). Bulk-heterojunction hybrid solar cells based on colloidal nanocrystals and conjugated polymers. Energy Environ. Sci.

[3] Wright M., Uddin A. (2012). Organic-inorganic hybrid solar cells: A comparative review. Solar Energy Mater. Solar Cells.

[4] Silva C. (2013). Organic Photovoltaics: Some like it hot. Nat. Mater.

[5] Deibel C., Strobel T., Dyakonov V. (2010). Role of the charge transfer state in organic donor-acceptor solar cells. Adv. Mater.

[6] Bansal N., Reynolds L.X., MacLachlan A., Lutz T., Ashraf R.S., Zhang W., Nielsen C.B., McCulloch I., Rebois D.G., Kirchartz T. (2013). Influence of crystallinity and energetics on charge separation in polymer-inorganic nanocomposite films for solar cells. Sci. Rep.

[7] Bhardwaj R.K., Kushwaha H.S., Gaur J., Upreti T., Bharti V., Gupta V., Chaudhary N., Sharma G.D., Banerjee K., Chand S. (2012). A green approach for direct growth of CdS nanoparticles network in poly(3-hexylthiophene-2,5-diyl) polymer film for hybrid photovoltaic. Mater. Lett.

[8] Cao X., Wang N., Kim X. (2011). Mesoporous CdS spheres for high-performance hybrid solar cells. Electrochim. Acta.

[9] Chen F., Qiu W.M., Chen X.Q., Yang L.G., Jiang X.X., Wang M., Chen H.Z. (2011). Large-scale fabrication of CdS nanorod arrays on transparent conductive substrates from aqueous solutions. Solar Energy.

[10] Cortina H., Martinez-Alonso C., Castillo-Ortega M., Hu H.L. (2012). Cellulose acetate fibers covered by CdS nanoparticles for hybrid solar cell applications. Mater. Sci. Eng. B Adv. Funct. Solid State Mater.

[11] Dimitriev O.P., Ogurtsov N.A., Pud A.A., Smertenko P.S., Piryatinski Y.P., Noskov Y.V., Kutsenko A.S., Shapoval G.S. (2008). Probing of charge and energy transfer in hybrid systems of aniline-3-methylthiophene copolymer with CdS and CdSe nanoparticles. J. Phys. Chem. C.

[12] Hu H.L., Kung S.C., Yang L.M., Nicho M.E., Penner R.M. (2009). Photovoltaic devices based on electrochemical-chemical deposited CdS and poly3-octylthiophene thin films. Solar Energy Mater. Solar Cells.

[13] Inpor K., Meeyoo V., Thanachayanont C. (2011). Enhancement of photovoltaic performance using hybrid CdS nanorods and MEH-PPV active layer in ITO/TiO_2_/MEH-PPV:CdS/Au devices. Curr. Appl. Phys.

[14] Lee J.C., Lee W., Han S.H., Kim T.G., Sung Y.M. (2009). Synthesis of hybrid solar cells using CdS nanowire array grown on conductive glass substrates. Electrochem. Commun.

[15] Li Y., Wang C.W., Zhao Y., Wang J., Zhou F. (2012). Performance improvement of P3HT/TiO_2_ coaxial heterojunction polymer solar cells by introducing a CdS interface modifier. J. Solid State Chem.

[16] Liao H.C., Chantarat N., Chen S.Y., Peng C.H. (2011). Annealing Effect on Photovoltaic Performance of Hybrid P3HT/In-Situ Grown CdS Nanocrystal Solar Cells. J. Electrochem. Soc.

[17] Masala S., Del Gobbo S., Borriello C., Bizzarro V., La Ferrara V., Re M., Pesce E., Minarini C., De Crescenzi M., Di Luccio T. (2011). Hybrid polymer-CdS solar cell active layers formed by *in situ* growth of CdS nanoparticles. J. Nanopart. Res.

[18] Wang L., Liu Y.S., Jiang X., Qin D.H., Cao Y. (2007). Enhancement of photovoltaic characteristics using a suitable solvent in hybrid polymer/multiarmed CdS nanorods solar cells. J. Phys. Chem. C.

[19] Wu S.J., Li J.H., Lo S.C., Tai Q.D., Yan F. (2012). Enhanced performance of hybrid solar cells based on ordered electrospun ZnO nanofibers modified with CdS on the surface. Org. Electron.

[20] Greaney M.J., Das S., Webber D.H., Bradforth S.E., Brutchey R.L. (2012). Improving Open Circuit Potential in Hybrid P3HT:CdSe Bulk Heterojunction Solar Cells via Colloidal tert-Butylthiol Ligand Exchange. Acs Nano.

[21] Schierhorn M., Boettcher S.W., Peet J.H., Matioli E., Bazan G.C., Stucky G.D., Moskovits M. (2010). CdSe Nanorods Dominate Photocurrent of Hybrid CdSe-P3HT Photovoltaic Cell. Acs Nano.

[22] Kim S., Kim D., Kim H.J., Lee Y.J., Hwang S.S., Baek K.Y., Ruck B. J., Kemmitt T. (2012). Hybrid Nanocomposite of CdSe Quantum Dots and a P3HT-b-PDMAEMA Block Copolymer for Photovoltaic Applications. Advanced Materials and Nanotechnology.

[23] Zhou Y.F., Riehle F.S., Yuan Y., Schleiermacher H.F., Niggemann M., Urban G.A., Kruger M. (2010). Improved efficiency of hybrid solar cells based on non-ligand-exchanged CdSe quantum dots and poly(3-hexylthiophene). Appl. Phys. Lett.

[24] Stalder R., Xie D.P., Zhou R.J., Xue J.G., Reynolds J.R., Schanze K.S. (2012). Variable-Gap Conjugated Oligomers Grafted to CdSe Nanocrystals. Chem. Mater.

[25] Zhang Q.L., Russell T.P., Emrick T. (2007). Synthesis and characterization of CdSe nanorods functionalized with regioregular poly(3-hexylthiophene). Chem. Mater.

[26] Zotti G., Vercelli B., Berlin A., Pasini M., Nelson T.L., McCullough R.D., Virgili T. (2010). Self-Assembled Structures of Semiconductor Nanocrystals and Polymers for Photovoltaics. 2. Multilayers of CdSe Nanocrystals and Oligo(poly)thiophene-Based Molecules. Optical, Electrochemical, Photoelectrochemical, and Photoconductive Properties. Chem. Mater.

[27] Dimitriev O.P., Ogurtsov N.A., Li Y.Q., Pud A.A., Gigli G., Smertenko P.S., Piryatinski Y.P., Noskov Y.V., Kutsenko A.S. (2012). Tuning of the charge and energy transfer in ternary CdSe/poly(3-methylthiophene)/poly(3-hexylthiophene) nanocomposite system. Colloid Polym. Sci.

[28] Park Y., Park J., Therien M.J., Stiff-Roberts A.D. (2012). Enhanced dispersion of CdSe/MEH-CN-PPV hybrid nanocomposites by *in situ* polymerization using AEM as photopolymerizable precursor. Colloid Polym. Sci.

[29] Jiu T.G., Reiss P., Guillerez S., de Bettignies R., Bailly S., Chandezon F. (2010). Hybrid Solar Cells Based on Blends of CdSe Nanorods and Poly(3-alkylthiophene) Nanofibers. IEEE J. Sel. Top. Quantum Electron.

[30] Brandenburg J.E., Jin X., Kruszynska M., Ohland J., Kolny-Olesiak J., Riedel I., Borchert H., Parisi J. (2011). Influence of particle size in hybrid solar cells composed of CdSe nanocrystals and poly(3-hexylthiophene). J. Appl. Phys.

[31] Saha S.K., Guchhait A., Pal A.J. (2012). Organic/inorganic hybrid pn-junction between copper phthalocyanine and CdSe quantum dot layers as solar cells. J. Appl. Phys.

[32] de Freitas J.N., Grova I.R., Akcelrud L.C., Arici E., Sariciftci N.S., Nogueira A.F. (2010). The effects of CdSe incorporation into bulk heterojunction solar cells. J. Mater. Chem.

[33] Qian L., Yang J.H., Zhou R.J., Tang A.W., Zheng Y., Tseng T.K., Bera D., Xue J.G., Holloway P.H. (2011). Hybrid polymer-CdSe solar cells with a ZnO nanoparticle buffer layer for improved efficiency and lifetime. J. Mater. Chem.

[34] Kwon S., Moon H.C., Lim K.G., Bae D., Jang S., Shin J., Park J., Lee T.W., Kim J.K. (2013). Improvement of power conversion efficiency of P3HT:CdSe hybrid solar cells by enhanced interconnection of CdSe nanorods via decomposable selenourea. J. Mater. Chem. A.

[35] Pokrop R., Pamula K., Deja-Drogomirecka S., Zagorska M., Borysiuk J., Reiss P., Pron A. (2009). Electronic, Electrochemical, and Spectroelectrochemical Properties of Hybrid Materials Consisting of Carboxylic Acid Derivatives of Oligothiophene and CdSe Semiconductor Nanocrystals. J. Phys. Chem. C.

[36] Radychev N., Lokteva I., Witt F., Kolny-Olesiak J., Borchert H., Parisi J. (2011). Physical Origin of the Impact of Different Nanocrystal Surface Modifications on the Performance of CdSe/P3HT Hybrid Solar Cells. J. Phys. Chem. C.

[37] Virgili T., Calzolari A., Lopez I.S., Vercelli B., Zotti G., Catellani A., Ruini A., Tassone F. (2013). Charge Separation in the Hybrid CdSe Nanocrystal-Organic Interface: Role of the Ligands Studied by Ultrafast Spectroscopy and Density Functional Theory. J. Phys. Chem. C.

[38] Zhong H.Z., Zhou Y., Yang Y., Yang C.H., Li Y.F. (2007). Synthesis of type IICdTe-CdSe nanocrystal heterostructured multiple-branched rods and their photovoltaic applications. J. Phys. Chem. C.

[39] Xu J.J., Hu J.C., Liu X.F., Qiu X.H., Wei Z.X. (2009). Stepwise Self-Assembly of P3HT/CdSe Hybrid Nanowires with Enhanced Photoconductivity. Macromol. Rapid Commun.

[40] Kois J., Bereznev S., Gurevits J., Volobujeva O. (2013). Electrochemically synthesised CdSe nanofibers and pearl-chain nanostructures for photovoltaic applications. Mater. Lett.

[41] Dayal S., Kopidakis N., Olson D.C., Ginley D.S., Rumbles G. (2010). Photovoltaic Devices with a Low Band Gap Polymer and CdSe Nanostructures Exceeding 3% Efficiency. Nano Lett.

[42] Schierhorn M., Boettcher S.W., Kraemer S., Stucky G.D., Moskovits M. (2009). Photoelectrochemical Performance of CdSe Nanorod Arrays Grown on a Transparent Conducting Substrate. Nano Lett.

[43] Roiban L., Hartmann L., Fiore A., Djurado D., Chandezon F., Reiss P., Legrand J.F., Doyle S., Brinkmann M., Ersen O. (2012). Mapping the 3D distribution of CdSe nanocrystals in highly oriented and nanostructured hybrid P3HT-CdSe films grown by directional epitaxial crystallization. Nanoscale.

[44] Han L.L., Qin D.H., Jiang X., Liu Y.S., Wang L., Chen J.W., Cao Y. (2006). Synthesis of high quality zinc-blende CdSe nanocrystals and their application in hybrid solar cells. Nanotechnology.

[45] Lek J.Y., Lam Y.M., Niziol J., Marzec M. (2012). Understanding polycarbazole-based polymer: CdSe hybrid solar cells. Nanotechnology.

[46] Albero J., Martinez-Ferrero E., Ajuria J., Waldauf C., Pacios R., Palomares E. (2009). Photo-induced electron recombination dynamics in CdSe/P3HT hybrid heterojunctions. Phys. Chem. Chem. Phys.

[47] Fan Z.X., Zhang H., Yu W.L., Xing Z.Y., Wei H.T., Dong Q.F., Tian W.J., Yang B. (2011). Aqueous-Solution-Processed Hybrid Solar Cells from Poly(1,4-naphthalenevinylene) and CdTe Nanocrystals. ACS Appl. Mater. Interfaces.

[48] Trotzky S., Kolny-Olesiak J., Falke S.M., Hoyer T., Lienau C., Tuszynski W., Parisi J. (2008). Ligand removal from soluble CdTe nanocrystals evidenced by time-resolved photoluminescence spectroscopy. J. Phys. D Appl. Phys.

[49] Ye S.J., Shen C., Pang H., Wang J., Lu Y. (2011). CdTe/PEDOT-PSS hybrid microspheres: Facile fabrication and multiple-color pH-sensing. Polymer.

[50] Verma D., Rao A.R., Dutta V. (2009). Surfactant-free CdTe nanoparticles mixed MEH-PPV hybrid solar cell deposited by spin coating technique. Solar Energy Mater. Solar Cells.

[51] Seo J., Kim S.J., Kim W.J., Singh R., Samoc M., Cartwright A.N., Prasad P.N. (2009). Enhancement of the photovoltaic performance in PbS nanocrystal:P3HT hybrid composite devices by post-treatment-driven ligand exchange. Nanotechnology.

[52] Wang Z.J., Qu S.C., Zeng X.B., Zhang C.S., Shi M.J., Tan F.R., Wang Z.G., Liu J.P., Hou Y.B., Teng F. (2008). Synthesis of MDMO-PPV capped PbS quantum dots and their application to solar cells. Polymer.

[53] Walsh A. (2011). Effects of reduced dimensionality on the electronic structure and defect chemistry of semiconducting hybrid organic-inorganic PbS solids. Proc. R. Soc. A.

[54] Guchhait A., Rath A.K., Pal A.J. (2011). To make polymer: Quantum dot hybrid solar cells NIR-active by increasing diameter of PbS nanoparticles. Solar Energy Mater. Solar Cells.

[55] Gunes S., Fritz K.P., Neugebauer H., Sariciftci N.S., Kumar S., Scholes G.D. (2007). Hybrid solar cells using PbS nanoparticles. Solar Energy Mater. Solar Cells.

[56] Feng Y.Y., Yun D.Q., Zhang X.Q., Feng W. (2010). Solution-processed bulk heterojunction photovoltaic devices based on poly(2-methoxy,5-octoxy)-1,4-phenylenevinylene-multiwalled carbon nanotubes/PbSe quantum dots bilayer. Appl. Phys. Lett.

[57] Tan Z.N., Zhang W.Q., Qian D.P., Zheng H., Xiao S.Q., Yang Y.P., Zhu T., Xu J., Gao K.Y., Zhou S.X., Zhao X.Q. (2011). Efficient hybrid infrared solar cells based on P3HT and PbSe nanocrystal quantum dots. Energy, Environment and Biological Materials.

[58] Zhu T., Berger A., Tan Z.A., Cui D.H., Xu J., Khanchaitit P., Wang Q. (2008). Composition-limited spectral response of hybrid photovoltaic cells containing infrared PbSe nanocrystals. J. Appl. Phys.

[59] Jiang X.M., Schaller R.D., Lee S.B., Pietryga J.M., Klimov V.I., Zakhidov A.A. (2007). PbSe nanocrystal/conducting polymer solar cells with an infrared response to 2 micron. J. Mater. Res.

[60] Yun D.Q., Feng W., Wu H.C., Yoshino K. (2009). Efficient conjugated polymer-ZnSe and -PbSe nanocrystals hybrid photovoltaic cells through full solar spectrum utilization. Solar Energy Mater. Solar Cells.

[61] Baeten L., Conings B., Boyen H.G., D’Haen J., Hardy A., D’Olieslaeger M., Manca J.V., Van Bael M.K. (2011). Towards Efficient Hybrid Solar Cells Based on Fully Polymer Infiltrated ZnO Nanorod Arrays. Adv. Mater.

[62] Noori K., Giustino F. (2012). Ideal Energy-Level Alignment at the ZnO/P3HT Photovoltaic Interface. Adv. Funct. Mater.

[63] Sung Y.H., Liao W.P., Chen D.W., Wu C.T., Chang G.J., Wu J.J. (2012). Room-Temperature Tailoring of Vertical ZnO Nanoarchitecture Morphology for Efficient Hybrid Polymer Solar Cells. Adv. Funct. Mater.

[64] Oosterhout S.D., Koster L.J.A., van Bavel S.S., Loos J., Stenzel O., Thiedmann R., Schmidt V., Campo B., Cleij T.J., Lutzen L. (2011). Controlling the Morphology and Efficiency of Hybrid ZnO:Polythiophene Solar Cells Via Side Chain Functionalization. Adv. Energy Mater.

[65] Sanchez S., Berson S., Guillerez S., Levy-Clement C., Ivanova V. (2012). Toward High-Stability Inverted Polymer Solar Cells with an Electrodeposited ZnO Electron Transporting Layer. Adv. Energy Mater.

[66] Liu J.P., Wang S.S., Bian Z.Q., Shan M., Huang C.H. (2009). Organic/inorganic hybrid solar cells with vertically oriented ZnO nanowires. Appl. Phys. Lett.

[67] Nagata T., Oh S., Yamashita Y., Yoshikawa H., Ikeno N., Kobayashi K., Chikyow T., Wakayama Y. (2013). Photoelectron spectroscopic study of band alignment of polymer/ZnO photovoltaic device structure. Appl. Phys. Lett.

[68] Pal E., Seemann T., Zollmer V., Busse M., Dekany I. (2009). Hybrid ZnO/polymer thin films prepared by RF magnetron sputtering. Colloid Polym. Sci.

[69] Taleatu B.A., Makinde W.O., Eleruja M.A., Fasasi A.Y. (2013). Band alignment and charge transport characteristics of TPP/ZnO hybrid for photovoltaic cell potential. Curr. Appl. Phys.

[70] Wang M.Q., Lian Y.Q., Wang X.G. (2009). PPV/PVA/ZnO nanocomposite prepared by complex precursor method and its photovoltaic application. Curr. Appl. Phys.

[71] Sydorov D., Smertenko P., Piryatinski Y., Yoshida T., Pud A. (2012). Electrochemically assembled planar hybrid poly(3-methylthiophene)/ZnO nanostructured composites. Electrochim. Acta.

[72] Zeyada H.M., El-Nahass M.M., El-Zawawi I.K., El-Menyawy E.M. (2010). Characterization of 2-(2,3-dihydro-1,5-dimethyl-3-oxo-2-phenyl-1H-pyrazol-4-ylimino)-2-(4-ni trophenyl)acetonitrile and ZnO nano-crystallite structure thin films for application in solar cells. Eur. Phys. J. Appl. Phys.

[73] Oh S., Nagata T., Volk J., Wakayama Y. (2013). Improving the performance of inorganic-organic hybrid photovoltaic devices by uniform ordering of ZnO nanorods and near-atmospheric pressure nitrogen plasma treatment. J. Appl. Phys.

[74] Chen J.Y., Hsu F.C., Sung Y.M., Chen Y.F. (2012). Enhanced charge transport in hybrid polymer/ZnO-nanorod solar cells assisted by conductive small molecules. J. Mater. Chem.

[75] Liu J.P., Choy K.L., Hou X.H. (2011). Charge transport in flexible solar cells based on conjugated polymer and ZnO nanoparticulate thin films. J. Mater. Chem.

[76] Lloyd M.T., Prasankumar R.P., Sinclair M.B., Mayer A.C., Olson D.C., Hsu J.W.P. (2009). Impact of interfacial polymer morphology on photoexcitation dynamics and device performance in P3HT/ZnO heterojunctions. J. Mater. Chem.

[77] Ruankham P., Sagawa T., Sakaguchi H., Yoshikawa S. (2011). Vertically aligned ZnO nanorods doped with lithium for polymer solar cells: Defect related photovoltaic properties. J. Mater. Chem.

[78] Ji L.W., Shih W.S., Fang T.H., Wu C.Z., Peng S.M., Meen T.H. (2010). Preparation and characteristics of hybrid ZnO-polymer solar cells. J. Mater. Sci.

[79] Shin K.S., Park H.J., Kumar B., Kim K.H., Kim S.H., Kim S.W. (2012). Inverted Organic Solar Cells with ZnO Nanowalls Prepared Using Wet Chemical Etching in a KOH Solution. J. Nanosci. Nanotechnol.

[80] Briseno A.L., Holcombe T.W., Boukai A.I., Garnett E.C., Shelton S.W., Frechet J.J.M., Yang P.D. (2010). Oligo- and Polythiophene/ZnO Hybrid Nanowire Solar Cells. Nano Lett.

[81] Krebs F.C., Thomann Y., Thomann R., Andreasen J.W. (2008). A simple nanostructured polymer/ZnO hybrid solar cell—Preparation and operation in air. Nanotechnology.

[82] Lee T.H., Sue H.J., Cheng X. (2011). ZnO and conjugated polymer bulk heterojunction solar cells containing ZnO nanorod photoanode. Nanotechnology.

[83] Sai N., Leung K., Chelikowsky J.R. (2011). Hybrid density functional study of oligothiophene/ZnO interface for photovoltaics. Phys. Rev. B.

[84] Bhat S.V., Govindaraj A., Rao C.N.R. (2011). Hybrid solar cell based on P3HT-ZnO nanoparticle blend in the inverted device configuration. Solar Energy Mater. Solar Cells.

[85] Li F., Du Y.H., Chen Y.W., Chen L., Zhao J., Wang P.S. (2012). Direct application of P3HT-DOPO@ZnO nanocomposites in hybrid bulk heterojunction solar cells via grafting P3HT onto ZnO nanoparticles. Solar Energy Mater. Solar Cells.

[86] Hao Y.Z., Cao Y.H., Sun B., Zhang Y.H., Li Y.P., Xu D.S., Li X.L. (2012). Fabrication of Ordered One-dimensional P3HT/CdS/TiO_2_/ZnO Core-Shell Nanorod Array and Their Application to Hybrid Solar Cell. Acta Chim. Sin.

[87] Zhu R., Jiang C.Y., Liu B., Ramakrishna S. (2009). Highly Efficient Nanoporous TiO_2_-Polythiophene Hybrid Solar Cells Based on Interfacial Modification Using a Metal-Free Organic Dye. Adv. Mater.

[88] Mor G.K., Shankar K., Paulose M., Varghese O.K., Grimes C.A. (2007). High efficiency double heterojunction polymer photovoltaic cells using highly ordered TiO_2_ nanotube arrays. Appl. Phys. Lett.

[89] Ozdal T., Hames Y., Aslan E. (2012). A comparative study on TiO_2_ doped hybrid solar cells. Appl. Surf. Sci.

[90] Lan Y.W., Zhou L.Y., Tong Z.F., Pang Q., Wang F., Gong F.Z. (2011). Synthesis and characterization of polythiophene-modified TiO_2_ nanotube arrays. Bull. Mater. Sci.

[91] Wiranwetchayan O., Zhang Q.F., Zhou X.Y., Liang Z.Q., Singjai P., Cao G.Z. (2012). Impact of the Morphology of TiO_2_ Films as Cathode Buffer Layer on the Efficiency of Inverted-Structure Polymer Solar Cells. Chalcogenide Lett.

[92] Boucle J., Chyla S., Shaffer M.S.P., Durrant J.R., Bradley D.D.C., Nelson J. (2008). Hybrid bulk heterojunction solar cells based on blends of TiO_2_ nanorods and P3HT. Comptes Rendus Phys.

[93] Qiao Q.Q., Su L.Y., Beck J., McLeskey J.T. (2005). Characteristics of water-soluble polythiophene: TiO2 composite and its application in photovoltaics. J. Appl. Phys.

[94] Lu M.D., Yang S.M. (2009). Synthesis of poly(3-hexylthiophene) grafted TiO_2_ nanotube composite. J. Colloid Interface Sci.

[95] Tai Q.D., Zhao X.Z., Yan F. (2010). Hybrid solar cells based on poly(3-hexylthiophene) and electrospun TiO2 nanofibers with effective interface modification. J. Mater. Chem.

[96] Han Y.G., Wu G., Li H.G., Wang M., Chen H.Z. (2010). Highly efficient ultraviolet photodetectors based on TiO_2_ nanocrystal-polymer composites via wet processing. Nanotechnology.

[97] Zeng T.W., Lin Y.Y., Lo H.H., Chen C.W., Chen C.H., Liou S.C., Huang H.Y., Su W.F. (2006). A large interconnecting network within hybrid MEH-PPV/TiO_2_ nanorod photovoltaic devices. Nanotechnology.

[98] Wang M.Q., Wang X.G. (2008). PPV/TiO_2_ hybrid composites prepared from PPV precursor reaction in aqueous media and their application in solar cells. Polymer.

[99] Dong Q.F., Yu W.L., Li Z.F., Yao S.Y., Zhang X.Y., Yang B., Im C., Tian W.J. (2012). All-water-solution processed solar cells based on PPV and TiO2 nanocrystals. Solar Energy Mater. Solar Cells.

[100] Liu J.C., Wang W.L., Yu H.Z., Wu Z.L., Peng J.B., Cao Y. (2008). Surface ligand effects in MEH-PPV/TiO_2_ hybrid solar cells. Solar Energy Mater. Solar Cells.

[101] Moon S.J., Baranoff E., Zakeeruddin S.M., Yeh C.Y., Diau E.W.G., Gratzel M., Sivula K. (2011). Enhanced light harvesting in mesoporous TiO2/P3HT hybrid solar cells using a porphyrin dye. Chem. Commun.

[102] Johansson E.M.J., Scholin R., Siegbahn H., Hagfeldt A., Rensmo H. (2011). Energy level alignment in TiO2/dipole-molecule/P3HT interfaces. Chem. Phys. Lett.

[103] Park Y.R., Lee Y.J., Yu C.J., Kim J.H. (2010). Investigations of the polymer alignment, the nonradiative resonant energy transfer, and the photovoltaic response of poly(3-hexylthiophene)/TiO_2_ hybrid solar cells. J. Appl. Phys.

[104] Davis R.J., Lloyd M.T., Ferreira S.R., Bruzek M.J., Watkins S.E., Lindell L., Sehati P., Fahlman M., Anthony J.E., Hsu J.W.P. (2011). Determination of energy level alignment at interfaces of hybrid and organic solar cells under ambient environment. J. Mater. Chem.

[105] Gruber M., Stickler B.A., Trimmel G., Schurrer F., Zojer K. (2010). Impact of energy alignment and morphology on the efficiency in inorganic-organic hybrid solar cells. Org. Electron.

[106] Kanai Y., Wu Z.G., Grossman J.C. (2010). Charge separation in nanoscale photovoltaic materials: Recent insights from first-principles electronic structure theory. J. Mater. Chem.

[107] Scharber M.C., Wuhlbacher D., Koppe M., Denk P., Waldauf C., Heeger A.J., Brabec C.L. (2006). Design rules for donors in bulk-heterojunction solar cells—Towards 10% energy—conversion efficiency. Adv. Mater.

[108] Oosterhout S.D., Wienk M.M., van Bavel S.S., Thiedmann R., Koster L.J., Gilot J., Loos J., Schmidt V., Janssen R.A. (2009). The effect of three-dimensional morphology on the efficiency of hybrid polymer solar cells. Nat. Mater.

[109] Liu R.C. (2009). Imaging of Photoinduced Interfacial Charge Separation in Conjugated Polymer/Semiconductor Nanocomposites. J. Phys. Chem. C.

[110] Hammer N.I., Emrick T., Barnes M.D. (2007). Quantum dots coordinated with conjugated organic ligands: New nanomaterials with novel photophysics. Nanoscale Res. Lett.

[111] Jeltsch K.F., Schadel M., Bonekamp J.B., Niyamakom P., Rauscher F., Lademann H.W.A., Dumsch I., Allard S., Scherf U., Meerholz K. (2012). Efficiency Enhanced Hybrid Solar Cells Using a Blend of Quantum Dots and Nanorods. Adv. Funct. Mater.

[112] Kanemoto R., Anas A., Matsumoto Y., Ueji R., Itoh T., Baba Y., Nakanishi S., Shikawa M., Biju V. (2008). Relations between dewetting of polymer thin films and phase-separation of encompassed quantum dots. J. Phys. Chem. C.

[113] Kim M., Shin M.J., Gwon D., Ahn H.S., Yi S.N., Kim P.S., Yoon S.C., Lee C., Park J., Shin K. (2013). Development of the Hybrid Conjugated Polymer Solar Cell Based on GaN Quantum Dots. Jpn. J. Appl. Phys.

[114] Lu H.W., Bao D.D., Penchev M., Ghazinejad M., Vullev V.I., Ozkan C.S., Ozkan M. (2010). Pyridine-Coated Lead Sulfide Quantum Dots for Polymer Hybrid Photovoltaic Devices. Adv. Sci. Lett.

[115] Nam M., Lee S., Park J., Kim S.W., Lee K.K. (2011). Development of Hybrid Photovoltaic Cells by Incorporating CuInS2 Quantum Dots into Organic Photoactive Layers. Jpn. J. Appl. Phys.

[116] Tan F.R., Qu S.C., Wu J., Liu K., Zhou S.Y., Wang Z.G. (2011). Preparation of SnS_2_ colloidal quantum dots and their application in organic/inorganic hybrid solar cells. Nanoscale Res. Lett.

[117] Greenham N.C., Peng X., Alivisatos A.P. (1996). Charge separation and transport in conjugated-polymer/semiconductor-nanocrystal composites studied by photoluminescence quenching and photoconductivity. Phys. Rev. B.

[118] Fang C., Qi X.Y., Fan Q.L., Wang L.H., Huang W. (2007). A facile route to semiconductor nanocrystal-semiconducting polymer complex using amine-functionalized rod-coil triblock copolymer as multidentate ligand. Nanotechnology.

[119] Landi B.J., Castro S.L., Ruf H.J., Evans C.M., Bailey S.G., Raffaelle R.P. (2005). CdSe quantum dot-single wall carbon nanotube complexes for polymeric solar cells. Solar Energy Mater. Solar Cells.

[120] Seo J., Kim W.J., Kim S.J., Lee K.S., Cartwright A.N., Prasad P.N. (2009). Polymer nanocomposite photovoltaics utilizing CdSe nanocrystals capped with a thermally cleavable solubilizing ligand. Appl. Phys. Lett.

[121] Stavrinadis A., Beal R., Smith J.M., Assender H.E., Watt A.A.R. (2008). Direct formation of PbS nanorods in a conjugated polymer. Adv. Mater.

[122] Zhao L., Pang X., Adhikary R., Petrich J.W., Jeffries-El M., Lin Z. (2011). Organic-inorganic nanocomposites by placing conjugated polymers in intimate contact with quantum rods. Adv. Mater.

[123] Reynolds L.X., Lutz T., Dowland S., MacLachlan A., King S., Haque S.A. (2012). Charge photogeneration in hybrid solar cells: A comparison between quantum dots and in situ grown CdS. Nanoscale.

[124] Reiss P., Couderc E., De Girolamo J., Pron A. (2011). Conjugated polymers/semiconductor nanocrystals hybrid materials-preparation, electrical transport properties and applications. Nanoscale.

[125] Moule A.J., Chang L.L., Thambidurai C., Vidu R., Stroeve P. (2012). Hybrid solar cells: Basic principles and the role of ligands. J. Mater. Chem.

[126] Gao F., Ren S.Q., Wang J.P. (2013). The renaissance of hybrid solar cells: Progresses, challenges, and perspectives. Energy Environ. Sci.

[127] Martinez-Ferrero E., Albero J., Palomares E. (2010). Materials, Nanomorphology, and Interfacial Charge Transfer Reactions in Quantum Dot/Polymer Solar Cell Devices. J. Phys. Chem. Lett.

[128] Zhao L., Lin Z.Q. (2012). Crafting Semiconductor Organic-Inorganic Nanocomposites via Placing Conjugated Polymers in Intimate Contact with Nanocrystals for Hybrid Solar Cells. Adv. Mater.

[129] Ruankham P., Macaraig L., Sagawa T., Nakazumi H., Yoshikawa S. (2011). Surface Modification of ZnO Nanorods with Small Organic Molecular Dyes for Polymer-Inorganic Hybrid Solar Cells. J. Phys. Chem. C.

[130] Saarenpaa H., Sariola-Leikas E., Perros A.P., Kontio J.M., Efimov A., Hayashi H., Lipsanen H., Imahori H., Lemmetyinen H., Tkachenko N.V. (2012). Self-Assembled Porphyrins on Modified Zinc Oxide Nanorods: Development of Model Systems for Inorganic-Organic Semiconductor Interface Studies. J. Phys. Chem. C.

[131] Said A.J., Poize G., Martini C., Ferry D., Marine W., Giorgio S., Fages F., Hocq J., Boucle J., Nelson J. (2010). Hybrid Bulk Heterojunction Solar Cells Based on P3HT and Porphyrin-Modified ZnO Nanorods. J. Phys. Chem. C.

[132] Sun B.Q., Marx E., Greenham N.C. (2003). Photovoltaic devices using blends of branched CdSe nanoparticles and conjugated polymers. Nano Lett.

[133] Lee K.S., Kim I., Gullapalli S., Wong M.S., Jabbour G.E. (2011). Enhanced performance of hybrid solar cells using longer arms of quantum cadmium selenide tetrapods. Appl. Phys. Lett.

[134] Huynh W.U., Dittmer J.J., Alivisatos A.P. (2002). Hybrid nanorod-polymer solar cells. Science.

[135] Soci C., Hwang I.W., Moses D., Zhu Z., Waller D., Gaudiana R., Brabec C.J., Heeger A.J. (2007). Photoconductivity of a low-bandgap conjugated polymer. Adv. Funct. Mater.

[136] Ren S., Chang L.Y., Lim S.K., Zhao J., Smith M., Zhao N., Bulovic V., Bawendi M., Gradecak S. (2011). Inorganic-organic hybrid solar cell: Bridging quantum dots to conjugated polymer nanowires. Nano Lett.

[137] Violi I.L., Perez M.D., Fuertes M.C., Soler-Illia G. (2012). Highly Ordered, Accessible and Nanocrystalline Mesoporous TiO_2_ Thin Films on Transparent Conductive Substrates. ACS Appl. Mater. Interfaces.

[138] Coakley K.M., McGehee M.D. (2003). Photovoltaic cells made from conjugated polymers infiltrated into mesoporous titania. Appl. Phys. Lett.

[139] Lee H.J., Leventis H.C., Haque S.A., Torres T., Gratzel M., Nazeeruddin M.K. (2011). Panchromatic response composed of hybrid visible-light absorbing polymers and near-IR absorbing dyes for nanocrystalline TiO_2_-based solid-state solar cells. J. Power Sources.

[140] Zhong M., Yang D., Zhang J., Shi J.Y., Wang X.L., Li C. (2012). Improving the performance of CdS/P3HT hybrid inverted solar cells by interfacial modification. Solar Energy Mater. Solar Cells.

[141] Krumm M., Pawlitzek F., Weickert J., Schmidt-Mende L., Polarz S. (2012). Temperature-Stable and Optically Transparent Thin-Film Zinc Oxide Aerogel Electrodes As Model Systems for 3D Interpenetrating Organic-Inorganic Heterojunction Solar Cells. ACS Appl. Mater. Interfaces.

[142] De Freitas J.N., Korala L., Reynolds L.X., Haque S.A., Brock S.L., Nogueira A.F. (2012). Connecting the (quantum) dots: Towards hybrid photovoltaic devices based on chalcogenide gels. Phys. Chem. Chem. Phys.

[143] Bi D.Q., Wu F., Qu Q.Y., Yue W.J., Cui Q., Shen W., Chen R.Q., Liu C.W., Qiu Z.L., Wang M.T. (2011). Device Performance Related to Amphiphilic Modification at Charge Separation Interface in Hybrid Solar Cells with Vertically Aligned ZnO Nanorod Arrays. J. Phys. Chem. C.

[144] Choi H.W., Lee K.S., Alford T.L. (2012). Optimization of antireflective zinc oxide nanorod arrays on seedless substrate for bulk-heterojunction organic solar cells. Appl. Phys. Lett.

[145] Han Y.G., Fan C.C., Wu G., Chen H.Z., Wang M. (2011). Low-Temperature Solution Processed Utraviolet Photodetector Based on an Ordered TiO2 Nanorod Array-Polymer Hybrid. J. Phys. Chem. C.

[146] Lee J.H., Shin J.H., Song J.Y., Wang W.F., Schlaf R., Kim K.J., Yi Y. (2012). Interface Formation Between ZnO Nanorod Arrays and Polymers (PCBM and P3HT) for Organic Solar Cells. J. Phys. Chem. C.

[147] Lim E.L., Yap C.C., Yahaya M., Salleh M.M. (2013). Enhancement of ZnO nanorod arrays-based inverted type hybrid organic solar cell using spin-coated Eosin-Y. Semicond. Sci. Technol.

[148] Nan Y.X., Chen F., Yang L.G., Jiang X.X., Zuo L.J., Zhang J.L., Yan Q.X., Shi M.M., Chen H.Z. (2011). Photoluminescence enhancement and atmosphere-dependent photovoltaic performance in CdS nanorod arrays/MEH-PPV hybrid. Solar Energy Mater. Solar Cells.

[149] Reinhard M., Conradt J., Braun M., Colsmann A., Lemmer U., Kalt H. (2012). Zinc oxide nanorod arrays hydrothermally grown on a highly conductive polymer for inverted polymer solar cells. Synth. Met.

[150] Takanezawa K., Hirota K., Wei Q.S., Tajima K., Hashimoto K. (2007). Efficient charge collection with ZnO nanorod array in hybrid photovoltaic devices. J. Phys. Chem. C.

[151] Takanezawa K., Tajima K., Hashimoto K. (2008). Charge Separation Interfaces in Polymer Photovoltaic Devices Hybridized with ZnO Nanorod Arrays. Jpn. J. Appl. Phys.

[152] Thitima R., Patcharee C., Takashi S., Susumu Y. (2009). Efficient electron transfers in ZnO nanorod arrays with N719 dye for hybrid solar cells. Solid State Electron.

[153] Tong F., Kim K., Martinez D., Thapa R., Ahyi A., Williams J., Kim D.J., Lee S., Lim E., Lee K.K., Park M. (2012). Flexible organic/inorganic hybrid solar cells based on conjugated polymer and ZnO nanorod array. Semicond. Sci. Technol.

[154] Yang C.Y., Liu S., Li M.R., Wang X.L., Zhu J., Chong R.F., Yang D., Zhang W.H., Li C. (2013). The role of glutathione on shape control and photoelectrical property of cadmium sulfide nanorod arrays. J. Colloid Interface Sci.

[155] Yuan Z.L., Yu J.S., Wang N.N., Jiang Y.D. (2011). Well-aligned ZnO nanorod arrays from diameter-controlled growth and their application in inverted polymer solar cell. J. Mater. Sci. Mater. Electron.

[156] Yun D.Q., Xia X.Y., Zhang S., Bian Z.Q., Liu R.H., Huang C.H. (2011). ZnO nanorod arrays with different densities in hybrid photovoltaic devices: Fabrication and the density effect on performance. Chem. Phys. Lett.

[157] Lee Y.J., Lloyd M.T., Olson D.C., Grubbs R.K., Lu P., Davis R.J., Voigt J.A., Hsu J.W.P. (2009). Optimization of ZnO Nanorod Array Morphology for Hybrid Photovoltaic Devices. J. Phys. Chem. C.

[158] Cui Q., Liu C.W., Wu F., Yue W.J., Qiu Z.L., Zhang H., Gao F., Shen W., Wang M.T. (2013). Performance Improvement in Polymer/ZnO Nanoarray Hybrid Solar Cells by Formation of ZnO/CdS-Core/Shell Heterostructures. J. Phys. Chem. C.

[159] Bi D.Q., Wu F., Yue W.J., Guo Y., Shen W., Peng R.X., Wu H.A., Wang X.K., Wang M.T. (2010). Device Performance Correlated with Structural Properties of Vertically Aligned Nanorod Arrays in Polymer/ZnO Solar Cells. J. Phys. Chem. C.

[160] Green M.A., Emery K., Hishikawa Y., Warta W., Dunlop E.D. (2012). Solar cell efficiency tables (version 40). Prog. Photovolt.

[161] Yu B.Y., Tsai A., Tsai S.P., Wong K.T., Yang Y., Chu C.W., Shyue J.J. (2008). Efficient inverted solar cells using TiO_2_ nanotube arrays. Nanotechnology.

[162] Wisnet A., Thomann M., Weickert J., Schmidt-Mende L., Scheu C. (2012). Nanoscale investigation on large crystallites in TiO_2_ nanotube arrays and implications for high-quality hybrid photodiodes. J. Mater. Sci.

[163] Shankar K., Mor G.K., Paulose M., Varghese O.K., Grimes C.A. (2008). Effect of device geometry on the performance of TiO2 nanotube array-organic semiconductor double heterojunction solar cells. J. Non Cryst. Solids.

[164] Rattanavoravipa T., Sagawa T., Yoshikawa S. (2008). Photovoltaic performance of hybrid solar cell with TiO_2_ nanotubes arrays fabricated through liquid deposition using ZnO template. Solar Energy Mater. Solar Cells.

[165] Mor G.K., Kim S., Paulose M., Varghese O.K., Shankar K., Basham J., Grimes C.A. (2009). Visible to Near-infrared Light Harvesting in TiO2 Nanotube Array-P3HT Based Heterojunction Solar Cells. Nano Lett.

[166] Lee J., Jho J.Y. (2011). Fabrication of highly ordered and vertically oriented TiO_2_ nanotube arrays for ordered heterojunction polymer/inorganic hybrid solar cell. Solar Energy Mater. Solar Cells.

[167] Hao Y.Z., Cao Y.H., Sun B., Li Y.P., Zhang Y.H., Xu D.S. (2012). A novel semiconductor-sensitized solar cell based on P3HT@CdS@TiO2 core-shell nanotube array. Solar Energy Mater. Solar Cells.

[168] Foong T.R.B., Shen Y.D., Hu X., Sellinger A. (2010). Template-Directed Liquid ALD Growth of TiO_2_ Nanotube Arrays: Properties and Potential in Photovoltaic Devices. Adv. Funct. Mater.

[169] Kim S., Mor G.K., Paulose M., Varghese O.K., Shankar K., Grimes C.A. (2010). Broad Spectrum Light Harvesting in TiO_2_ Nanotube Array—Hemicyanine Dye-P3HT Hybrid Solid-State Solar Cells. IEEE J. Sel. Top. Quantum Electron.

[170] Janaky C., Bencsik G., Racz A., Visy C., de Tacconi N.R., Chanmanee W., Rajeshwar K. (2010). Electrochemical Grafting of Poly(3,4-ethylenedioxythiophene) into a Titanium Dioxide Nanotube Host Network. Langmuir.

[171] Thelese R.B.F., Chan K.L., Hu X. (2012). Structure and properties of nano-confined poly(3-hexylthiophene) in nano-array/polymer hybrid ordered-bulk heterojunction solar cells. Nanoscale.

[172] Byun J., Kim Y., Jeon G., Kim J.K. (2011). Ultrahigh Density Array of Free-Standing Poly(3-hexylthiophene) Nanotubes on Conducting Substrates via Solution Wetting. Macromolecules.

[173] Rath T., Edler M., Haas W., Fischereder A., Moscher S., Schenk A., Trattnig R., Sezen M., Mauthner G., Pein A. (2011). A Direct Route Towards Polymer/Copper Indium Sulfide Nanocomposite Solar Cells. Adv. Energy Mater.

[174] Liu Z., Sun Y., Yuan J., Wei H., Huang X., Han L., Wang W., Wang H., Ma W. (2013). High-efficiency hybrid solar cells based on polymer/PbS*_x_* Se_1−_*_x_* nanocrystals benefiting from vertical phase segregation. Adv. Mater.

[175] Chen Z.L., Zhang H., Du X.H., Cheng X., Chen X.G., Jiang Y.Y., Yang B. (2013). From planar-heterojunction to n-i structure: An efficient strategy to improve short-circuit current and power conversion efficiency of aqueous-solution-processed hybrid solar cells. Energy Environ. Sci.

[176] Zhou R.J., Stalder R., Xie D.P., Cao W.R., Zheng Y., Yang Y.X., Plaisant M., Holloway P.H., Schanze K.S., Reynolds J.R., Xue J.G. (2013). Enhancing the Efficiency of Solution-Processed Polymer: Colloidal Nanocrystal Hybrid Photovoltaic Cells Using Ethanedithiol Treatment. ACS Nano.

[177] Zhou R.J., Xue J.G. (2012). Hybrid Polymer-Nanocrystal Materials for Photovoltaic Applications. Chemphyschem.

[178] Saunders B.R. (2012). Hybrid polymer/nanoparticle solar cells: Preparation, principles and challenges. J. Colloid Interface Sci.

